# A systematic review and meta-analysis of endocrine-related adverse events associated with interferon

**DOI:** 10.3389/fendo.2022.949003

**Published:** 2022-08-05

**Authors:** Linghuan Wang, Binqi Li, He Zhao, Peixin Wu, Qingzhen Wu, Kang Chen, Yiming Mu

**Affiliations:** ^1^ Medicine School of Nankai University, Tianjin, China; ^2^ Department of Endocrinology, Chinese People’s Liberation Army (PLA) General Hospital, Beijing, China

**Keywords:** interferon, endocrine adverse events, hypothyroidism, hyperthyroidism, thyroiditis

## Abstract

**Objectives:**

To perform a systematic review and meta-analysis of interferon and endocrine side effects, including their incidence, evaluation, and management.

**Methods:**

PubMed was searched through March 7th, 2021, by 2 authors independently (LH Wang and H Zhao). Early phase I/II, phase III experimental trials, prospective and retrospective observational studies were included. Stata 16.0 (StataCorp LLC, 16.0) was the main statistical software for meta-analysis. The weighted incidence and risk ratio were estimated for primary thyroid disease and diabetes mellitus.

**Results:**

A total of 108 studies involving 46265 patients were included. Hypothyroidism was the most common thyroid disorder, followed by hyperthyroidism. IFN α+RBV treated patients experienced hypothyroidism in 7.8% (95%CI, 5.9-9.9), which was higher than IFN α (5.2%; 95%CI, 3.7-6.8) and IFN β (7.0%; 95%CI, 0.06-23.92). IFN α+RBV treated patients experienced hyperthyroidism in 5.0% (95%CI, 3.6-6.5), which was higher than IFN α (3.5%; 95%CI, 2.5-4.8) and IFN β (3.4%; 95%CI, 0.9-7.5). The summary estimated incidence of painless thyroiditis was 5.8% (95%CI, 2.8-9.8) for IFN α, and 3.5% (95%CI,1.9-5.5) for IFN α+RBV. The summary estimated incidence of diabetes was 1.4% (95%CI, 0.3-3.1) for IFN, 0.55% (95%CI, 0.05-1.57) for IFN α, 3.3% (95%CI,1.1-6.6) for IFN α+RBV.

**Conclusions:**

Our meta-analysis shows a high incidence of endocrine adverse events provoked by IFN, further reinforced by combined RBV treatment.

**Systematic Review Registration:**

https://www.crd.york.ac.uk/prospero/, identifier CRD42022334131.

## Introduction

Interferon (IFN) is a broad-spectrum antiviral agent that activates cell surface receptors and causes cells to produce antiviral proteins, thereby inhibiting viral replication and interferon use has long been associated with endocrine-related adverse events (1). About 0.1 to 1% of patients suffered severe and even life-threatening side effects, including thyroid, visual, hearing, kidney, and heart damage, and pulmonary interstitial fibrosis (2, 3), in which, thyroid disorders have been reported in up to 20% of the patients during IFN-based therapies (4). Besides, cases of diabetes mellitus associated with interferon have also been reported. The present study aims to perform a systematic review and meta-analysis of interferon and endocrine side effects, including their incidence, evaluation, and management.

## Materials and methods

### Study registration

The review has been registered with the International Prospective Systems Review Registry (PROSPERO ID: CRD42022334131).

### Literature search

Two independent authors (LH Wang and H Zhao) searched PubMed database for relevant articles on the subject of endocrinopathies and interferon until March 7th, 2021. Search terms were included for the various endocrinopathies, adverse events, interferon, IFN, hypothyroidism, hyperthyroidism, thyroiditis, hypophysitis, primary adrenal insufficiency, diabetes, Graves’ disease, primary thyroid disease, thyroid dysfunction, secondary hypogonadotropic hypogonadism, primary hypoparathyroidism, primary hypoparathyroidism. Combine these terms with the Boolean logic operator AND/OR.

### Study selection

Study design types included early phase I/II, phase III experimental trials, prospective, and retrospective observational studies. The subjects were adults treated with interferon. Studies of interferon combined with radiotherapy, cellular vaccines, small molecule inhibitors, immune checkpoint inhibitors (ICI), or interleukin-2 therapy regimen were excluded. The language was limited to English or Chinese. A preliminary selection of manuscripts was made by title and abstract. Then, studies that did not report adverse endocrine events or had inadequate data were excluded by reading the full text. In addition, duplicate studies were excluded. If there was any disagreement, it would be discussed by all authors and resolved by consensus.

### Data analysis and extraction

Each included study included the following elements: author and year of publication, study design, median follow-up time, therapy, dosing of drug administration, endocrine adverse events (hypothyroidism, hyperthyroidism, thyroiditis, and diabetes mellitus). Supplementary data and appendices were also methodically explored if available.

### Outcome

The outcome was the occurrence of endocrine adverse events (such as hypothyroidism, hyperthyroidism, and diabetes mellitus).

### Definition of hypothyroidism, hyperthyroidism, painless thyroiditis and diabetes

Hypothyroidism was defined as an elevated TSH with a decreased fT4 and/or fT3 level. Hyperthyroidism was defined as a suppressed TSH with an elevated fT4 and/or fT3 level. Painless thyroiditis was defined as hyperthyroidism or hypothyroidism secondary to thyrotoxicosis, with negative TRAb, reduced or absent uptake of technetium scan tracer, and/or increased 18 fluorodeoxyglucose uptake of positron emission tomography (18FDG-PET) tracer (5). Thyroid biopsy was a better way to diagnose painless thyroiditis but it was invasive. Therefore, PET was used to help diagnose painless thyroiditis. Diabetes was defined as FBG ≥ 7.0 mmol/L, and/or PBG ≥ 11.1 mmol/L.

### Statistical analysis

Stata 16.0 (StataCorp LLC, 16.0) was the main statistical software for meta-analysis. A meta-analysis of incidence estimates was performed using an inverse sine transformation method to weigh the studies. Summary estimates of incidence were reported with a 95% confidence interval (CI). For randomized controlled trials, relative risk and 95% CI were calculated using the number of adverse events observed and the number of patients who did not experience an adverse event in each group. In addition, we conducted a combination of the relative risks from individual trials for the same adverse event in a meta-analysis. Heterogeneity was assessed using the Q and I² statistics. Where there was significant statistical heterogeneity, we reported risk ratios (RRs) using the random-effects model. For other outcomes, we used the fixed-effect model. P<0.05 was considered statistically significant.

## Results

### Study characteristics

**Figure 1:**
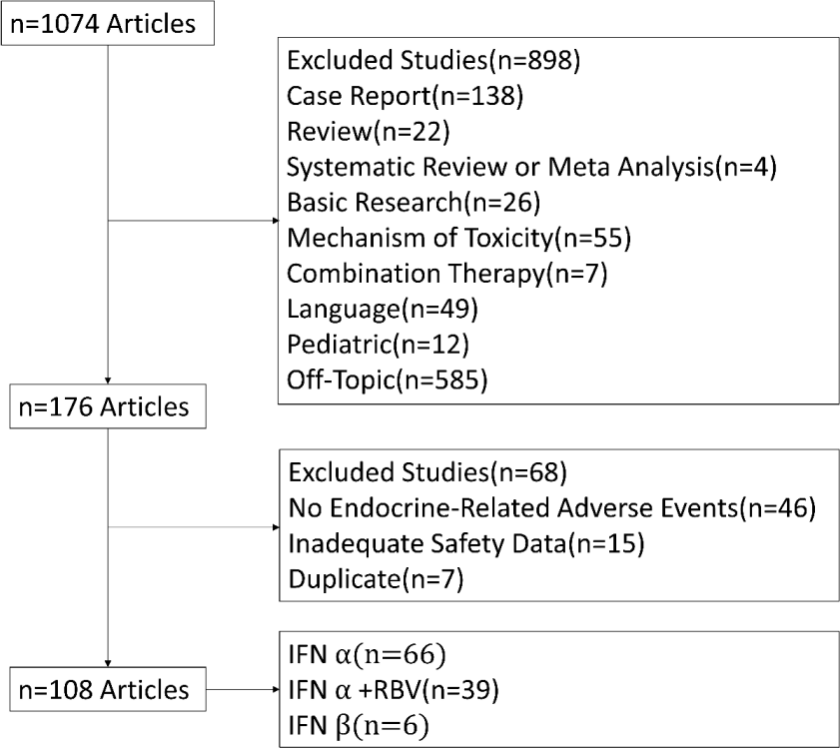
Flow chart of study selection.

Our search identified a total of 1074 articles, of which 898 were excluded based on title and abstract. Of the 176 full texts that were reviewed, 108 were finally included (**Figure 1**) (2, 6–89). These included 1 phase I, 6 phase III randomized trials, 75 prospective, and 26 retrospective studies. A total of 119 study arms were identified. The main diseases were chronic hepatitis C (CHC) (82/119, 68.9%), chronic hepatitis B (CHB) (13/119, 10.9%), CHB+CHC (2/119, 1.7%), chronic hepatitis D (2/119, 1.7%), multiple sclerosis (7/119, 5.9%), malignant tumor (9/119, 7.6%) and blood disease (4/119, 3.4%). A total of 46265 patients were analyzed (IFN α, n=21344; IFN β, n=1625; IFN α+RBV, n=11937; placebo, n=10204). The regimens were classified as monotherapy with IFN α (70/119, 58.8%), IFN β (7/119, 5.9%), and IFN α+RBV (43/119, 36.1%).

### Incidence of endocrine adverse events

The summary estimated incidence of endocrine adverse events was 9.1% (95%CI, 8.7-10.7) for IFN, 8.3% (95%CI, 6.4-10.4) for IFN α, 8.3% (95%CI,2.9-16.2) for IFN β, 12.7% (95%CI,10-15.7) for IFN α+RBV (**Table 1**).

**Table 1:** Summary estimated incidence of endocrine adverse events on interferon.

		Endocrine adverse events		Hypothyroidism		Hyperthyroidism		Diabetes mellitus	
	Total patients	Analyzed patients	Summary incidence	Analyzed patients	Summary incidence	Analyzed patients	Summary incidence	Analyzed patients	Summary incidence
Treatment	n	N (%)	%(95%CI)	N (%)	%(95%CI)	N (%)	%(95%CI)	N (%)	%(95%CI)
IFN	36061	35322 (98.0)	9.1 (8.7-10.7)	32146 (89.1)	5.2 (4.2-6.3)	30727 (85.2)	3.6 (2.8-4.4)	12586 (34.9)	1.4 (0.3-3.1)
IFN α	21344	20886 (97.9)	8.3 (6.4-10.4)	19915 (93.3)	5.2 (3.7-6.8)	18785 (88.0)	3.5 (2.5-4.8)	12313 (57.7)	0.55 (0.05-1.57)
IFN β	1625	1625 (100)	8.3 (2.9-16.2)	854 (52.6)	7.0 (0.06-23.92)	1002(61.7)	3.4 (0.9-7.5)	NR	NR
IFN α+RBV	11937	11825 (99.1)	12.7 (10-15.7)	10754 (90.1)	7.8 (5.9-9.9)	10581 (88.6)	5.0 (3.6-6.5)	273 (2.3)	3.3 (1.1-6.6)

The number of analyzed patients (%) is also reported. NR, Not reported.

### Incidence of hypothyroidism

Hypothyroidism was the most common thyroid disorder. The summary estimated incidence of hypothyroidism was 5.2% (95%CI, 4.2-6.3) for IFN, 5.2% (95%CI, 3.7-6.8) for IFN α, 7.0% (95%CI,0.06-23.92) for IFN β, 7.8% (95%CI,5.9-9.9) for IFN α+RBV. Compared to placebo, the risk of hypothyroidism was significantly increased by IFN (RR, 1.999; 95% CI, 1.043–3.831; p =0.037). However, there was no difference between IFN α (RR, 2.634; 95% CI, 0.559–11.588; p >0.05)/IFN α+RBV (RR, 1.861; 95% CI, 0.647–5.350; p >0.05) and placebo (**Tables 1**, **2**).

**Table 2:** ** **Summary of relative risk for endocrine adverse events.

	Hypothyroidism		Hyperthyroidism	
Treatment	% (95%CI)	p-value	% (95%CI)	p-value
IFN vs. Placebo	1.999 (1.043-3.831)	0.037	2.329 (1.667-3.253)	<0.001
IFN α vs. Placebo	2.634 (0.559-11.588	>0.05	2.145 (0.348-13.216)	>0.05
IFN α+RBV vs. Placebo	1.861 (0.647-5.350)	>0.05	2.319 (1.647-3.266)	<0.001
IFN β vs. Placebo	NR	NR	NR	NR

NR, Not reported.

### Incidence of hyperthyroidism

Predictions for hyperthyroidism were lower with a combined incidence of 3.6% (95%CI, 2.8-4.4) for IFN, 3.5% (95%CI, 2.5-4.8) for IFN α, 3.4% (95%CI, 0.9-7.5) for IFN β, 5.0% (95%CI, 3.6-6.5) for IFN α+RBV. Compared to placebo, the risk of hyperthyroidism was significantly increased by IFN (RR, 2.329; 95% CI, 1.667–3.253; p <0.001). The IFN α+RBV (RR, 2.319; 95% CI, 1.647–3.266; p < 0.001) had a high risk of hyperthyroidism compared to the placebo. However, there was no difference between IFN α (RR, 2.145; 95% CI, 0.348–13.216; p >0.05) and placebo (**Tables 1**, **2**).

### Incidence of painless thyroiditis

The summary estimated incidence of painless thyroiditis was 3.7% (95%CI, 2.3-5.3) for IFN, 5.8% (95%CI, 2.8-9.8) for IFN α, 3.5% (95%CI, 1.9-5.5) for IFN α+RBV. Compared to placebo, the risk of painless thyroiditis was significantly increased by IFN (RR, 1.854; 95% CI, 1.270–2.707; p=0.001). The IFN α+RBV (RR, 1.875; 95% CI, 1.281–2.745; p = 0.001) had a high risk of painless thyroiditis compared to the placebo. Patients with painless thyroiditis can be detected both in the phase of hyperthyroidism or hypothyroidism, further complicating correct reporting. It was not appropriate to put painless thyroiditis and other endocrine adverse events in one table. Therefore, painless thyroiditis was analyzed separately (**Tables S1**, **S2**).

### Incidence of diabetes mellitus

The summary estimated incidence of diabetes mellitus was 1.4% (95%CI, 0.3-3.1) for IFN, 0.55% (95%CI, 0.05-1.57) for IFN α, 3.3% (95%CI,1.1-6.6) for IFN α+RBV. Further analysis was not possible due to the rarity of interferon-associated diabetes (**Table 1**).

## Discussion

Our meta-analysis shows a high incidence of endocrine adverse events related to interferon therapy, which is further enhanced by combined therapy. Hypothyroidism is the most common thyroid disorder, followed by hyperthyroidism. The highest incidence of hypothyroidism on monotherapy is noted on IFN β. The incidence of hyperthyroidism during monotherapy is higher for IFN α. Diabetes mellitus are less frequent, with no cases of diabetes mellitus reported on IFN β therapy. IFN α+RBV shows a remarkably higher incidence of hypothyroidism, hyperthyroidism, and diabetes mellitus. However, IFN α+RBV shows a lower incidence of painless thyroiditis than IFN α.

### Thyroid dysfunction

Interferon is a broad-spectrum antiviral drug, which is widely used in the treatment of viral hepatitis and neoplastic diseases. Interferon therapy is closely associated with the occurrence of thyroid dysfunction (TD).

The reported incidence of interferon-associated thyroid dysfunction ranged from 2.5% to 34.3% (9, 14–16, 18, 76, 90, 91, 40, 69, 92–94). In addition, the incidence of interferon-associated TD was correlated with the duration of treatment. Cumulative incidence increased with the duration of interferon therapy, with 6.6%, 10.1%, and 11.5% cumulative incidence at 3, 6, and 12 months after initiation of interferon treatment, respectively (69).

Different treatments had different effects on the incidence of thyroid dysfunction. The incidence of thyroid dysfunction during IFN α combined with RBV was reported to be 4.7 to 27.8% (14–16, 93, 95). The incidence of thyroid dysfunction during INF α monotherapy was 2.5%.-34.3% (14, 40, 94). Besides, studies found that patients treated with interferon α and ribavirin had a higher mean incidence of TD (12.1%) than patients treated with interferon α alone (6.6%) (96).

The incidence of hypothyroidism, hyperthyroidism, and thyroiditis during interferon-α therapy was 4.2 - 6.4%, 1.0 - 5.1%, and 1.7 - 3.4% respectively (14, 69). Previous studies have shown varying timing of onset of thyroid dysfunction during interferon therapy. The mean time to onset of thyroid dysfunction after interferon treatment was 5-6 months (14, 69), among which, the average onset time of hypothyroidism was 5.1 weeks to 6 months (3, 14), the average onset time of hyperthyroidism was 6.8 weeks to 4 months (3, 14), and the average onset time of thyroiditis was 18 weeks to 4 months (3, 14).

However, the long-term outcome of these patients who suffered interferon-associated thyroid dysfunction remains unknown. Most prospective studies were limited to 6 months after the end of IFN α therapy (92). Most TD patients presented with subclinical thyroid disease, which spontaneously recovered after interferon treatment, and only a few patients had persistent symptoms that required medication (3, 14, 69, 95). However, Vasiliadis et al. reported that 57.69% of patients displayed permanent thyroid disease (22). Besides, thyroid disease may even persist in some patients, requiring long-term treatment (18, 23, 94).

The incidence of IFN α associated with TD also differed significantly between countries. In two studies of adult subjects, Brazil had the lowest incidence and Poland had the highest (25, 97).

In our study, the proportion of hypothyroidism was higher than that of hyperthyroidism, as in most studies (9, 14, 22, 92). Contrary to our results, a study in southern Taiwan found that hyperthyroidism was most common in interferon alpha-based therapy (23).

So why are there differences in the incidence of interferon-associated TD reported by different studies? First, difference in genetic susceptibility was one of the leading factors of TD incidence variability. Studies showed that Asian ethnicity was an independent risk factor for IFN-associated TD development, with a higher incidence than other ethnic groups (27, 76). Second, iodine status also played an important role in TD variation. A diet high in iodine was prone to hypothyroidism, and a diet high in iodine was prone to hyperthyroidism. Third, different definitions of TD may overestimate or underestimate the true prevalence of TD.

IFN caused disorders of the immune system, and it had a direct toxic effect on thyroid cells. IFN induced cytotoxicity by upregulating perforin expression in peripheral natural killer cells and T cells, especially T helper cells (Th). It suppressed Th2 and enhanced Th1 immune response (91). Besides, interferon also activated lymphocytes and made cytokine and thyroid antibody production increase (91). IFN directly disrupted the thyroid. IFN inhibited hormone production, secretion, and metabolism, and lead to abnormal expression of major histocompatibility antigens on thyroid cells (29, 98). In addition to IFN α, ribavirin also had immunomodulatory effects on the thyroid (99). What’s more, HCV itself also induced thyroid autoantibodies (100, 101).

Previous studies showed that gender was an independent factor in predicting the occurrence of TD, and females had an increased risk for the development of IFN associated TD (9, 18, 23, 27, 76, 102). We believed that the hormonal status of women was one of the reasons why women were more susceptible to IFN-α related TD than men. The immune reactivity of females was higher than that of males, and sex hormones affected the occurrence and severity of immune-mediated pathological states. In addition, studies found that pretreatment of TPOAb was an independent factor associated with TD occurrence, and patients with positive pretreatment of TPOAb had a higher risk of TD occurrence (22, 35, 36, 38, 76). What’s more, ATA positive rate also predict TD (35, 40). Studies showed that HCV patients with positive ATA had an 80% chance of developing TD during or after interferon therapy. The prevalence of ATA in patients with hepatitis C was significantly higher than that in healthy people (22, 35, 90, 101, 103).

### Diabetes mellitus

Interferon-related diabetes was also a major endocrine adverse event. Although the incidence was not high, the presentation was usually severe and irreversible, with fulminant diabetes and ketoacidosis (104, 105). In one of our previous reviews, we found that interferon therapy shortened the incubation period of T2DM, turning the original T2DM into T1DM (106). The onset of interferon-associated type 1 diabetes [0.50 (0.55) years] required longer periods of IFN treatment than interferon-associated type 2 diabetes [0.19 (0.28) years] (106). The incidence of T1DM in hepatitis C patients treated with interferon was 10-18 times that of the general population (2, 43, 104). It was worth noting that interferon-induced diabetes may be accompanied by the occurrence of autoimmune thyroid disease or changes in immune indicators (107–117).

IFN was significantly overexpressed in islet cells of T1DM patients (118–120). Interferon may contribute to diabetes in several ways. First, IFN α lead to apoptosis by activating oligosadenosine synthase-ribonuclease L and protein kinase R pathways (121). Second, Interferon-alpha increased membrane known major histocompatibility complexIclass antigen expression and activated T cells and natural killer cells causingβ-cell injury (122). Third, Th1 multiactivity induced by IFN α therapy enhanced the autoimmune response to β cells and accelerated the destruction of β cells (123). Compared with non-peg-IFN, patients receiving the combination of peg-IFN and ribavirin had a shorter time to develop IFN-induced T1DM (124). The incidence of diabetes mellitus was higher for IFN α+RBV than for IFN α in our study. Ribavirin was a guanosine analog that had an immune effect on TH1-like activation (125), which further enhanced the autoimmune response to interferon. On the one hand, Th1 cytokines expressed Fas antigen on the surface of β cells and induced T-cell mediated apoptosis (68, 126). On the other hand, as a Th1 cytokine, IFN γ directly damaged β cells by synergistic action with TFN α (127).

### Other endocrine events

Few data are available on thyroid cancer and gonadal function during interferon therapy. In one prospective review, one interferon-alpha-treated patient was identified with papillary thyroid carcinoma (71). Besides, a prospective study of interferon therapy for hepatitis from China reported a case of gonadal dysfunction.

### Precision medicine and future directions

Interferon can also cause other autoimmune diseases, including optic neuromyelitis (128), Sjogren’s syndrome (114), severe insulin resistance against insulin receptor antibodies (109, 113), and stiff-man syndrome, in addition to causing endocrine adverse events. However, unlike targeted therapies (129), reports of interferon-related adverse events toward the skin and mucous manifestations were rare. Some interferon-associated adverse endocrine events were severe and irreversible, such as type 1 diabetes and stiff-man syndrome. Therefore, it was necessary to strengthen the monitoring of high risk factors (e.g., thyroid related antibodies, diabetes related antibodies, other autoimmune antibodies) during interferon therapy. Aggressive treatment was also necessary for interferon-associated endocrine adverse events to avoid more serious consequences.

Currently, the main treatment drugs for chronic viral hepatitis include nucleoside/nucleotide analogue (NUC) drugs and immune stimulants such as interferon α (IFN α) or PEG-IFN α. However, more advanced treatments such as gene therapy, targeted immune therapy (130) and modern vaccination are now emerging rapidly (131). In addition, precision medicine is picking up pace. Pharmacogenomics Studies such as Human Genome Project, genome-wide association studies (GWAS) and other pharmacogenomics studies (132), Next-Generation Gene Sequencing and Mass Spectrometric Studies (133) are the main content of precision medicine. In addition, the design and creation of advanced diagnostic tools and nanomedicine Precision Therapy (PTAs) maximizes the detection, treatment and monitoring of chronic viral hepatitis (134, 135).

### Study design and limitations

This is a comprehensive analysis of endocrine adverse events on interferon. Because adverse events are often underestimated in randomized clinical trials, our meta-analysis includes retrospective, prospective studies, and randomized clinical trials. Differences in the definition of thyroid disease such as hypothyroidism, hyperthyroidism, and thyroiditis in each study may lead to differences in its incidence. Besides, subclinical thyroid disease has been reported as an adverse event in some studies and not in others, which may have a great impact on morbidity. In addition, our analysis was conducted at the study level and did not include data from individual patients. Human error can’t be excluded in the screening of included studies. Finally, the trial will be excluded if endocrine adverse events are not reported, which may overestimate our final results.

## Conclusions

Thyroid diseases are frequent endocrine adverse events triggered by interferon, and hypothyroidism is most common. Combined ribavirin shows a remarkably higher incidence. Besides, interferon-related diabetes is also a major endocrine adverse event. Although the incidence is not high, the presentation is usually severe. Therefore, clinically, a high suspicion of adverse endocrine events is necessary. Timely diagnosis and treatment of adverse endocrine events can avoid life-threatening complications. Precision medicine will be an advanced treatment for chronic viral hepatitis.

## Data availability statement

The original contributions presented in the study are included in the article/**Supplementary Material**. Further inquiries can be directed to the corresponding authors.

## Author contributions

LW performed the statistical analysis and interpreted the data, drafted and revised the manuscript. LW, KC, and YM contributed to design the conception of the manuscript. BL, HZ, PW, QW, KC, and YM revised the manuscript. Every author contributed in the final approval of the version to be published and agreement to be accountable for all aspects of the work in ensuring that questions related to the accuracy or integrity of any part of the work are appropriately investigated and resolved.

## Funding

Funding resources was provided by the Beijing Municipal Science & Technology Commission (Project No. D141107005314004) and the Beijing Municipal Science & Technology Commission (Project No.Z201100005520014).

## Acknowledgments

We thank Professor Yiming Mu and Professor Kang Chen for their direction on this analysis.

## Conflict of interest

The authors declare that the research was conducted in the absence of any commercial or financial relationships that could be construed as a potential conflict of interest.

## Publisher’s note

All claims expressed in this article are solely those of the authors and do not necessarily represent those of their affiliated organizations, or those of the publisher, the editors and the reviewers. Any product that may be evaluated in this article, or claim that may be made by its manufacturer, is not guaranteed or endorsed by the publisher.
