# Supplementary material for: A systematic review and meta-analysis of endocrine-related adverse events associated with interferon
Source: Front Endocrinol (Lausanne). 2022 Aug 5;13:949003. doi: 10.3389/fendo.2022.949003 (PMC9388759; doi:10.3389/fendo.2022.949003)
Supplement: Supplementary file 1 [file Table_1.docx]

**Table s1 Summary estimated incidence of painless thyroiditis on interferon**

|  |  | Silent thyroiditis | |
| --- | --- | --- | --- |
|  | Total patients | Analyzed patients | Summary incidence |
| Treatment | n | N (%) | %(95%CI) |
| IFN | 36061 | 22776(63.2) | 3.7(2.3-5.3) |
| IFN α | 21344 | 14207(66.6) | 5.8(2.8-9.8) |
| IFN β | 1625 | NR | NR |
| IFN α+RBV | 11937 | 7704(64.5) | 3.5(1.9-5.5) |

The number of analyzed patients (%) is also reported. NR: Not reported.

**Table s2 Summary of relative risk for painless thyroiditis**

|  | Thyroiditis | |
| --- | --- | --- |
| Treatment | %(95%CI） | p-value |
| IFN vs. Placebo | 1.854(1.270-2.707) | 0.001 |
| IFN α vs. Placebo | NR | NR |
| IFN α+RBV vs. Placebo | 1.875(1.281-2.745) | 0.001 |
| IFN β vs. Placebo | NR | NR |

NR: Not reported.
